# A Method for Inferring an Individual’s Genetic Ancestry and Degree of Admixture Associated with Six Major Continental Populations

**DOI:** 10.3389/fgene.2012.00322

**Published:** 2013-01-14

**Authors:** Ondrej Libiger, Nicholas J. Schork

**Affiliations:** ^1^Department of Molecular and Experimental Medicine, The Scripps Research Institute and the Scripps Translational Science InstituteLa Jolla, CA, USA

**Keywords:** genetic ancestry, admixture, population genetics, admixture proportions

## Abstract

The determination of the ancestry and genetic backgrounds of the subjects in genetic and general epidemiology studies is a crucial component in the analysis of relevant outcomes or associations. Although there are many methods for differentiating ancestral subgroups among individuals based on genetic markers only a few of these methods provide actual estimates of the fraction of an individual’s genome that is likely to be associated with different ancestral populations. We propose a method for assigning ancestry that works in stages to refine estimates of ancestral population contributions to individual genomes. The method leverages genotype data in the public domain obtained from individuals with known ancestries. Although we showcase the method in the assessment of ancestral genome proportions leveraging largely continental populations, the strategy can be used for assessing within-continent or more subtle ancestral origins with the appropriate data.

## Introduction

Allele frequencies at most loci throughout the genome vary among populations (Cavalli-Sforza et al., 1994). Although the within-population variance in allele frequencies is much higher than between-population variance (Lewontin, 1973), individuals can be grouped into clusters that correspond to major world populations based on alleles they possess at multiple loci (Edwards, 2003). Dense genotyping data can thus be used to estimate an individual’s biogeographic ancestry. Individuals with recent ancestors that originated in different populations will inevitably show mixed membership in several ancestral clusters, and the degree to which they can be considered members of one ancestral group or another is an indication of the degree to which their genome was derived from different ancestral populations (Rosenberg et al., 2002).

As noted, most techniques used for assessing variation in genetic background and ancestry among a sample of individuals based on the observed genotypic profiles those individuals possess rely on “unsupervised” clustering approaches, whereby individuals in a sample with similar genotypic profiles are considered members of a particular ancestral group whose origins or geographic and historical context is not immediately obvious (Pritchard et al., 2000; Tang et al., 2005; Alexander et al., 2009). These approaches are well suited for the identification of genetically homogeneous subgroups in a data set as well as for quantification of the genetic variability within the dataset. However, a number of research efforts require, or would significantly benefit from, describing each individual’s biogeographic ancestry in the context of the known global biogeographical populations. For example, it may be of value to know whether an individual’s genotypic profile is more consistent with that individual’s ancestors originating in a European, African, or possibly European/African admixed population. The majority of studies that require this information use self-reported ancestry as a proxy for biogeographic ancestry. However, this practice has many limitations (Pfaff et al., 2001; Klimentidis et al., 2009; Tayo et al., 2011), especially for recently admixed individuals, such as Hispanics or African Americans, whose genetic ancestry has been shaped by admixture from several source continental populations, and the precise contribution from each source population is often unknown. In the case of African Americans, for example, it is often useful to determine the degree of African ancestry for each individual (i.e., that individual’s admixture proportions). Such information can be used to provide a detailed yet intuitive description of the individual’s genetic ancestry, which could be important for correcting for population substructure in association studies, since it has been shown that ignoring ancestral and genetic background heterogeneity in a study investigating associations between phenotypes and/or genotypes among a group of individuals can lead to both false positive and false negative results (Li, 1972; Lander and Schork, 1994; Cardon and Palmer, 2003; Marchini et al., 2004; Platt et al., 2010; Price et al., 2010). In addition, individual ancestry estimation is necessary for relating phenotypes to the variation in genetic background (Allison et al., 2010; Fejerman et al., 2010; Kumar et al., 2010; Yang et al., 2011), as well as developing appropriate reference panels for, e.g., determining population-specific allele frequencies or searching for *de novo* mutations that are unlikely to occur in other individuals within a given population (Solovieff et al., 2010).

Detailed information regarding admixture may also be useful in identifying genomic regions that have undergone recent selection (Grossman et al., 2010; Johnson et al., 2011). A number of studies have shown that, despite their sharing ancestries, recently admixed individuals exhibit large variation in admixture proportions associated with ancestral continental populations (Parra et al., 1998; Bertoni et al., 2003; Bonilla et al., 2004; Sinha et al., 2006; Via et al., 2011), and thus would pose a challenge for analyses that employ unsupervised clustering techniques to assess their genetic ancestry (Johnson et al., 2011).

Several relevant research efforts have focused on identifying a small set of ancestry informative markers (AIMs’) that can be used to infer biogeographic ancestry and admixture proportions (Parra et al., 1998; Collins-Schramm et al., 2004; Enoch et al., 2006; Tian et al., 2006; Galanter et al., 2012). However, the majority of AIM panels are designed to determine admixture proportions between only two or three source populations, and are thus often intended to be used for individuals with a specific population history such as African Americans or Hispanics. In addition, AIM panels often trade off accuracy of ancestry estimates for genotyping speed and low costs by including only a limited number of highly informative markers. Since, allele frequencies at a vast majority of loci in the genome differ among major continental populations, albeit slightly, resolving biogeographic ancestry with greater accuracy requires the use of genotype data at many loci (Price et al., 2010). Given continued reductions in genotyping costs, it is quite likely that whole genome SNP array data or whole genome sequencing data will be available for ancestry estimation purposes in the future, leading to very accurate estimates of individual admixture proportions.

We propose using genotype data on a set of reference individuals with known biogeographic ancestry associated with six major continental groups to generate accurate, relevant, and easily interpretable admixture proportions for individuals. To this end, we constructed a reference panel from publicly available data, and developed a methodology that utilizes this panel to provide admixture proportions associated with African, Central Asian, East Asian, European, Native American, and Oceanic populations. Individuals from five of these populations (all but Central Asia) were previously found to form well-defined clusters that could be distinguished reliably with a panel of genetic markers (Rosenberg et al., 2002, 2005). Thus, we were confident that with this reference set of individuals, we could reliably estimate an individual’s ancestry relative to these six major continental groups. We devised a two-step procedure to obtain accurate admixture estimates. The first step involves running a supervised analysis with bootstrapping implemented in the ADMIXTURE software (Alexander et al., 2009; Alexander and Lange, 2011) with the proposed reference panel and a set of target individuals whose ancestry is to be determined as input. In the second step, we utilize the standard errors associated with the initial admixture proportion estimates computed via bootstrapping in the first step to reduce the number of ancestral populations likely to contribute to each individual, with the aim of refining the initial admixture proportions. We performed a resampling study that assessed the validity of the proposed reference panel, and also assessed the accuracy of the proposed two-step method by comparing the estimated admixture proportions obtained from the procedure with known admixture proportions based on parental information for a group of offspring.

## Materials and Methods

### Reference panel construction

We constructed a reference panel of individuals from six major continental populations by gathering genotype data collected for 2513 individuals of known ancestry from 83 populations around the world using several publicly available sources, including the Human Genome Diversity Project (HGDP, Cann et al., 2002), the Population Reference (POPRES, Nelson et al., 2008), HapMap3 (Altshuler et al., 2010), and the University of Utah dataset (Xing et al., 2009). To obtain reference individuals that uniquely capture genetic variation from the six major continental populations, the reference panel was created in a stepwise fashion in order to ensure that the individuals included do not exhibit admixture across the six continental populations, and that each continental population is represented by a reasonably large number of diverse individuals originating in the relevant continent. To do this, we first collated data for all 1350 European, 527 African, and 64 Native American individuals (1941 in total), and clustered this set into three arbitrary clusters based on allele frequency differences using unsupervised ADMIXTURE analysis (Alexander et al., 2009). Note that we used the software ADMIXTURE to perform model-based clustering in the development of the reference panel as well as in all subsequent analyses. This program estimates individual admixture proportions from multi-locus SNP data using a maximum-likelihood method. It employs a similar statistical model as the program STRUCTURE (Pritchard et al., 2000) but uses fast numerical optimization algorithm to achieve greater speed, and is therefore suitable for supervised clustering of genome-wide genotype data collected on a large numbers of individuals.

Although most individuals clustered together with other individuals of the same documented ancestry, this was not always the case. We removed individuals with an estimated admixture proportion of <0.9 associated with their “correct” previously assigned continental cluster. Note that we explored the impact of using different cutoffs, and concluded that the 0.9 cutoff was close to optimal for our purposes; for example, a more stringent cutoff of 0.95 resulted in the exclusion of entire populations such as the Maya, which is one of the few representatives of Central American Native American people in the publicly available data, and a significant source of admixture in many contemporary Mexican individuals. Exactly 1748 individuals remained in the reference panel representing every European, African, and Native American population that is represented in the public sources except the Maasai, who were all excluded based on their imperfect clustering. All Hema individuals except one (out of 15) were also excluded. Interestingly, this analysis suggested that an individual labeled 14,374 in POPRES is, in contrast to this individual’s reported ancestry, of non-European descent, and this individual was also excluded from the reference panel.

In the second step, we incorporated genotype data from individuals of East Asian descent into the panel. Unsupervised ADMIXTURE analysis assuming four clusters did not yield clusters corresponding to the intended population units (likely due to the greater within-population differentiation among Africans compared to Eurasians). We therefore resorted to a supervised ADMIXTURE analysis (Alexander and Lange, 2011) with explicitly defined ancestry for all European, African, Native American as well as Japanese individuals (chosen arbitrarily to anchor the cluster of East Asian individuals), with the goal of estimating admixture proportions for the remaining East Asian individuals. This analysis identified 453 additional East Asian individuals who exhibited a >0.9 admixture proportion associated with the population cluster defined by the Japanese individuals. All Yakut individuals had to be excluded from the reference panel due to apparent admixture. Analogously, we explicitly defined the ancestry of all individuals included in the reference panel up to this point, and added Oceanic individuals from Melanesia and New Guinea. In order to create the basis for an additional cluster of “Oceanic” individuals, we explicitly defined the ancestry of a small number of Oceanic individuals. For a number of different subsets, Melanesian individuals consistently exhibited approximately 20% admixture with other population groups. For this reason, we excluded all Melanesian individuals from the reference panel, as well as individual HGDP00544 with a reported ancestry of New Guinea, who also seemed to be admixed. Thus, sixteen New Guinean individuals were added to the reference panel to represent the Oceanic population. In the final step, we added genotype data for Central Asian individuals, and ran a supervised ADMIXTURE analysis with explicitly defined ancestry for all non-Central Asian individuals as well as a small number of Punjabi individuals. Exactly 297 Central Asian individuals exhibited admixture proportion >0.9 associated with the Central Asian cluster defined by the Punjabi, and were added to the reference panel to represent Central Asia. Individual 15145 labeled as Urdu clustered clearly with European individuals and not Central Asians.

By the cumulative merging of all these data, we were able to assemble a reference panel containing genotype information at 16,433 strand-ambiguous SNPs positioned throughout the genomes of 2513 individuals from 63 populations spread across five continents (see Tables 1 and 2). The 16,433 SNPs reflected the maximum number of markers that were typed in common among all the individuals in the final reference panel. These markers exhibited low LD (*r*^2^ < 0.1 was observed between 99% of marker pairs) and allele frequency higher than 1%. The maximum proportion of individuals with missing genotypes per SNP was <5%, and the maximum proportion of missing genotypes per person was <0.01. By restricting individuals assigned to the six continental populations to those with >0.9 similarity to other individuals assigned to the same continental population, we have likely excluded individuals and population subgroups that lived in a particular continent but were recent immigrants to the continent. This is important for ancestry estimation since it ensures proper temporal ancestral relationships, not merely geographic relations. The reference panel dataset is available upon request from the authors.

**Table 1:** **Documented ancestry of the individuals contained in the reference panel**.

Europe	Albania	3
	Austria	13
	Basque	24
	Belgium	41
	Bergamo	13
	Bosnia	9
	Croatia	7
	Cyprus	4
	Czech Republic	9
	England	21
	France	114
	Germany	66
	Greece	7
	Hungary	19
	Ireland	61
	Italy	209
	Kosovo	15
	Macedonia	4
	Netherlands	16
	Orcadian	15
	Poland	22
	Portugal	133
	Romania	14
	Russia	16
	Sardinian	31
	Scotland	5
	Serbia	9
	Spain	131
	Sweden	9
	Swiss-French	99
	Swiss-German	83
	Swiss-Italian	11
	Tuscan	85
	Yugoslavia	17
	Total	1335
Africa	!Kung	16
	Alur	10
	Biaka Pygmies	22
	East Bantu	9
	Hema	1
	Luhya	86
	Mandenka	22
	Mbuti Pygmies	38
	Nguni	9
	Pedi	9
	Sotho/Tswana	7
	South Bantu	8
	Yoruba	129
	Total	366
America	Columbia	6
	Karitiana	14
	Maya	5
	Pima	14
	Surui	8
	Total	47
East Asia	Cambodian	3
	Dai	10
	Daur	3
	Han	129
	Hezhen	6
	Iban	24
	Japan	198
	Lahu	8
	Miaozu	10
	Mongola	7
	Naxi	8
	Oroqen	3
	She	10
	Tu	2
	Tujia	10
	Vietnamese	7
	Xibo	5
	Yizu	10
	Total	453
Oceania	New Guinea	16
Central Asia	Andhra Brahmin	25
	Balochi	1
	Dalit	1
	Gujarati	79
	Hindi	2
	Irula	1
	Pathan	1
	Punjabi	157
	Sindhi	1
	Tamil Brahmin	12
	Tamil in Sri Lanka	9
	Urdu	7
	Total	296
Total		2513

**Table 2:** **Fixation index (Fst – lower diagonal) and identity-by-state sharing-based distance (IBS – upper diagonal in italics) between pairs of major continental populations comprising the Reference Panel**.

	Europe	Africa	America	East Asia	Oceania	Central Asia
Europe	*0.28*	*0.34*	*0.32*	*0.32*	*0.33*	*0.29*
Africa	0.15	*0.27*	*0.36*	*0.34*	*0.35*	*0.34*
America	0.17	0.26	*0.24*	*0.3*	*0.33*	*0.32*
East Asia	0.11	0.18	0.12	*0.27*	*0.32*	*0.31*
Oceania	0.22	0.28	0.3	0.21	*0.22*	*0.32*
Central Asia	0.03	0.14	0.14	0.07	0.19	*0.29*

*IBS distance between two populations was calculated as the mean distance between all individuals from one population and all individuals from the other population. IBS distance for a single population (on the diagonal) was determined using the mean IBS distance between all pairs of different individuals from the population*.

### Denoising procedure

Estimating admixture proportions based on a finite sample of reference individuals and genetic markers necessarily produces estimates that exhibit a level of uncertainty due to a sampling error. However, this error can be estimated via simulation-based techniques such as bootstrapping (Kunsch, 1989). We developed a technique to reduce the noise associated with the admixture proportions by using the standard errors calculated for each individual’s degree of ancestry (or ancestral contribution) from each of the six continental populations to refine the admixture estimates. In this denoising approach, we first compute admixture proportion estimates for all individuals for each of the six continental populations using the entire set of reference individuals and determine the estimates’ standard errors via bootstrapping as implemented in ADMIXTURE (Alexander et al., 2009). We next use the subset of the six continental populations to estimate individual ancestry proportions that exhibited statistically significant evidence of contributing to an individual’s ancestry based on 95% confidence intervals of the relevant admixture proportions. In other words, for each individual whose ancestry is being determined, we subtract two times the standard error from the corresponding admixture proportion estimate associated with each one of the six continental populations. If the resulting value is smaller or equal to zero, we conclude that there is not sufficient evidence to conclude that the continental population in question contributed to the ancestry of the individual. We exclude a subset of the reference populations for which this is true from the subsequent supervised ADMIXTURE analysis aimed at refining the admixture proportions. The entire procedure, including the denoising process, takes approximately 1 min of computing time per target individual using a standard desktop computer. The python script is available upon request from the authors or at http://genomics.scripps.edu/ancestry/.

### Validation procures

In order to confirm that the genetic ancestry of the individuals in the assembled reference panel is representative of the continental population consistent with the individuals’ documented ancestry we leveraged two independent techniques: principal coordinate analysis (PCoA; Gower, 1966) and Generalized Analysis of Molecular Variance (GAMOVA; Nievergelt et al., 2007). PCoA is a data analysis method used to graphically display complex information regarding, e.g., the genetic dissimilarity of individuals in a lower dimensional space with the least possible loss of information. GAMOVA is a regression based technique used to quantify the strength of the association between a genetic dissimilarity matrix formed between pairs of individuals and grouping factors such as population membership. This technique utilizes the full similarity data without reducing the number of dimensions.

## Results

### Reference panel validity

**Figure 1:**
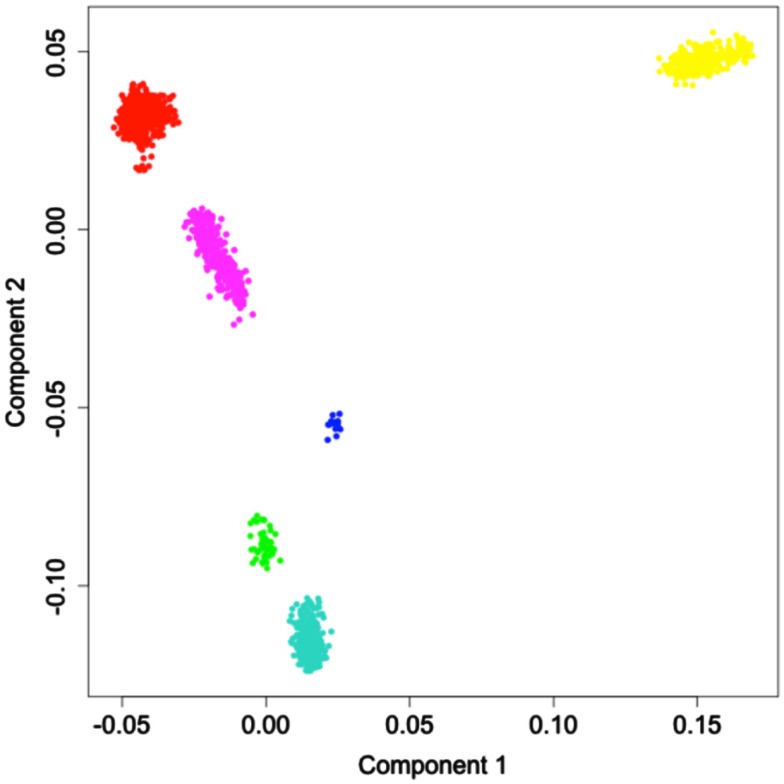
**Multidimensional scaling of the identity-by-state sharing dissimilarity between pairs of reference individuals**. Colors represent the major continental populations where the individuals originated. Red: Europe; Yellow: Africa; Green: America; Turquiose: East Asia; Blue: Oceania; Magenta: Central Asia.

The results of principal coordinate analysis based on identity-by-state sharing (IBS) matrix is shown in Figure 1. Each point in the plot represents an individual from the reference panel, and is colored according to the individual’s documented ancestry. Neighboring points represent individuals who are genetically more similar. As can be seen in Figure 1, these points form six distinct clusters. The uniform coloring of the points within each cluster suggests that these clusters represent discrete groups of individuals that originate from different continental populations. The projection used to generate Figure 1 reduced the multidimensional genetic dissimilarity data into two dimensions, which maximally explain the total genetic variability in the panel. In this case, the two dimensions accounted for approximately 17% of the variance in the dissimilarity, and each additional dimension explained <1% of the remaining variability.

GAMOVA analysis (Nievergelt et al., 2007) suggested that a highly statistically significant proportion of total variance in the genetic variation exhibited by the individuals in our reference panel was explained by grouping the individuals into each one of the six continental populations, thus providing statistical evidence (*p* < 0.001) that individuals within each one of the six populations are genetically more similar (in terms of their IBS sharing) compared to a set of individuals randomly chosen from the entire reference panel, regardless of their ancestry.

**Figure 2:**
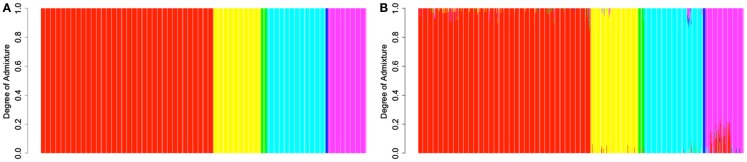
**(A)** Admixture proportions for individuals in the reference panel estimated by a supervised analysis using their documented ancestry information. **(B)** Admixture proportions estimated for each individual in the reference panel in a resampling analysis via the procedure proposed in this report. Red: Europe; Yellow: Africa; Green: America; Turquiose: East Asia; Blue: Oceania; Magenta: Central Asia. Individuals’ admixture proportions are presented in the same order in both panels.

**Figure 3:**
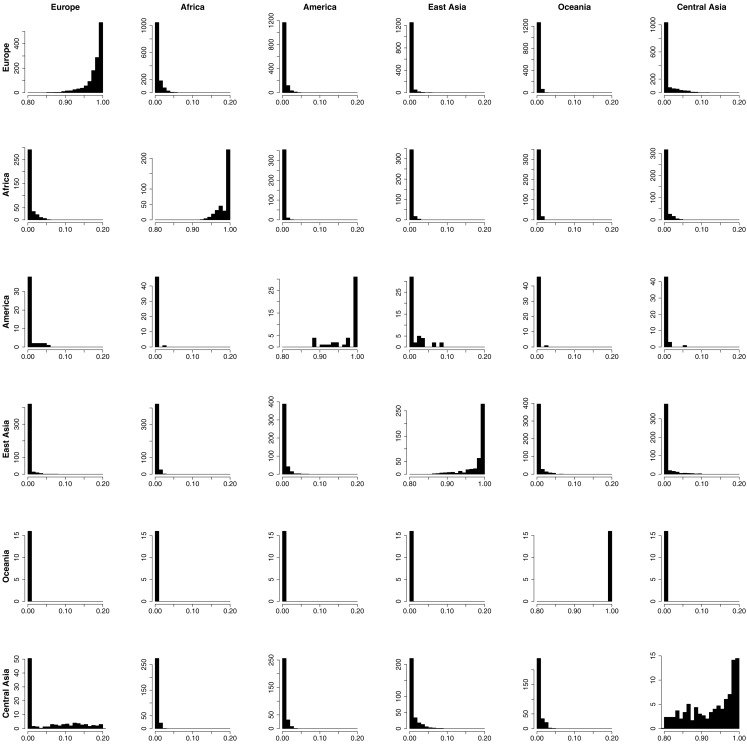
**The distribution of admixture from the six major continental populations (in columns) estimated for each individual in the Reference Panel in turn using a “leave-one-out” sequential analysis supervised by the documented ancestry of the remaining 2512 individuals**. The ancestry of the individual whose admixture proportions were being estimated in each step were presumed to be unknown.

In order to provide further evidence of the validity of the reference panel, and to demonstrate the utility of the proposed methodology in assigning admixture proportion to individuals of unknown ancestry, we carried out a resampling scheme designed to assess the ability of the proposed computational procedure to determine the ancestry of the reference individuals that is consistent with their documented ancestry. In each one of 2513 resampling iterations, we selected a single individual from the reference panel and assumed that his or her ancestry was unknown. Using genotype and ancestry information for the 2512 remaining individuals from the reference panel, we estimated the admixture proportions for the selected individual. We then compared the obtained admixture proportions to this individual’s documented ancestry. The estimated admixture proportions for each individual are shown in Figure 2B. Figure 2A shows the documented ancestry for the same individuals for comparison. This analysis suggests that the predominant source of admixture estimated by the proposed approach is in agreement with the documented ancestry of each individual. Figure 3 shows the admixture proportions estimated via the resampling procedure in greater detail. The histograms show the distribution of admixture proportions from the six major continental populations (in columns) for all reference individuals who share the same documented ancestry (in rows). Ideally, the histograms located along the main diagonal should show distributions centered around 1.0, while the off-diagonal histograms would present distributions centered around 0.0. The vast majority of European reference individuals were estimated to possess 95% or greater european admixture even though their documented European ancestry was not taken into account in the analysis (top left histogram). On the contrary, estimates for the vast majority of these individuals suggested the presence of <5% admixture from Central Asian populations. This analysis shows that the documented ancestry was recapitulated relatively less accurately for Central Asian individuals, although relevant admixture proportions for all Central Asian individuals were still higher than 70% (63% of these individuals exhibited proportions associated with Central Asian ancestry that were higher than 90%).

To further assess the reliability of the admixture proportions obtained by applying the proposed procedure, we simulated genotypes at 16,443 loci according to equation 1 in Alexander et al. (2009) for 100 admixed individuals. The simulated admixture proportions (*q*_i_) were set to 0.5 for European and 0.5 for Native American populations. Allele frequencies for these populations were estimated based on the described reference panel. After running our ancestry inference method, we obtained a mean admixture estimate of 0.49 (SD = 0.01) associated with European populations, and admixture estimate of 0.51 (SD = 0.01) associated with Native American populations. The mean contribution of the remaining four continental population was estimated to be smaller than 0.0005. We also simulated a three-way admixture in additional 100 individuals by setting admixture proportions to 0.2, 0.05, and 0.75 associated with European, Native American, and African populations respectively. In this analysis, our procedure yielded a mean African admixture estimate of 0.75 (SD = 0.01), European admixture estimate of 0.2 (SD = 0.01), and Native American estimate of 0.05 (SD = 0.01). Other continental populations were estimated to contribute <0.001 to the ancestry of these 100 simulated genomes. This simulation study suggests that for Mexican Americans and African Americans, our proposed methodology yields accurate admixture estimates.

### Application to available data sets

**Figure 4:**
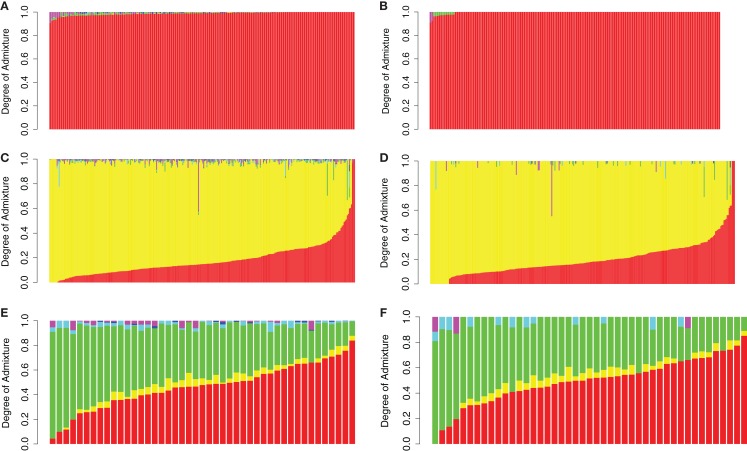
**Admixture proportions of European Americans (A,B), African Americans (C,D) and Mexican Americans (E,F) obtained via a supervised analysis using the Reference Panel before denoising (A,C,E) and after denoising (B,D,F)**. Individuals are sorted by degree of european admixture. Red: Europe; Yellow: Africa; Green:America; Turquiose: East Asia; Blue: Oceania; Magenta: Central Asia.

In order to demonstrate the utility of the reference panel and the proposed computational method, we assessed the admixture proportions in publicly available genotype datasets containing 161 European American, 292 African American, and 45 Mexican American individuals (Nelson et al., 2008; Xing et al., 2009; Altshuler et al., 2010). We essentially assumed that the ancestry of these individuals was unknown and applied our procedure to determine their ancestry. Consistent with expectations, we were able to classify the vast majority (148, 92%) of European Americans as having 100% European ancestry. Thirteen individuals (8%) documented as European Americans exhibited <10% of admixture from either Native American (11 individuals) or Central Asian (two individuals) populations (see Figure 4B). Figure 4A shows the results of this analysis before applying our denoising procedure, demonstrating the effect of the denoising procedure. The majority of African Americans exhibited between 40 and 90% of African ancestry with most of the remaining admixture emanating from European populations (Figures 4C,D). Admixture proportions for Mexican American individuals suggested the presence of various degrees of Native American and European admixture with a small degree of African admixture in some cases (Figures 4E,F). Although we are not able to determine the precision of these estimates because the true admixture proportions are unknown, these results are consistent with previous findings regarding genetic ancestry of African and Mexican Americans and with the known history of these populations (Parra et al., 1998; Bertoni et al., 2003; Bonilla et al., 2004; Gonzalez Burchard et al., 2005; Sinha et al., 2006; Hancock et al., 2009; Klimentidis et al., 2009; Johnson et al., 2011; Tayo et al., 2011).

### Reliability of the denoising procedure

**Figure 5:**
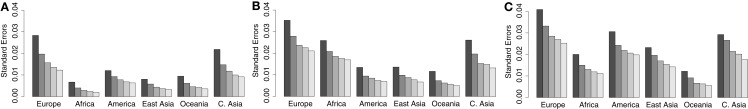
**Mean standard errors associated with admixture proportions for European Americans (A), African Americans (B), Mexican Americans (C) obtained via a supervised analysis using 3236, 6592, 9902, 13110, and 16443 SNPs (darkest to lightest bars)**.

The effects of the denoising procedure in eliminating small (and likely artifactual) admixture proportions from certain continental populations while emphasizing the contribution from continental populations with unequivocal statistical evidence of admixture are clearly apparent from Figure 4. This effect is also demonstrated by the data presented in Table 3, which lists the proportion of European American, African American, and Mexican American individuals, who exhibit >1% admixture from the six continental populations before and after denoising. However, a valid concern regarding the use of standard errors to refine the estimates in the denoising procedure is that if the standard errors are too high due to an unsufficient number of SNPs used in the estimation of the admixture proportions, the continental populations with low admixture proportions relative to the associated error may be incorrectly excluded from the subsequent analysis aimed a trefining the admixture estimates. To address this issue, we pursued additional resampling analyses to determine whether the proposed reference panel included a sufficient number of SNPs. We compared standard errors (used in the denoising procedure) associated with admixture estimates obtained for European Americans, African Americans, and Mexican Americans using a fraction of the genotype data available in the reference panel. The modified reference panel used in each analysis contained a randomly selected subset of the 16,433 total available SNPs for all 2513 individuals. The results are shown in Figure 5 and suggest that increasing the number of markers beyond the 16,433 SNPs that are currently included in the reference panel would not significantly decrease the standard errors associated with admixture proportions.

**Table 3:** **Percentage of individuals with more than 1% of estimated admixture contributed by the six major continental populations before and after denoising**.

	European Americans		African Americans		Mexican Americans	
	With noise (%)	Denoised (%)	With noise (%)	Denoised (%)	With noise (%)	Denoised (%)
Europe	100	100	97	94	100	98
Africa	1	0	99	99	91	80
America	29	7	32	10	100	100
East Asia	1	0	20	4	76	27
Oceania	6	0	10	3	18	0
Central Asia	11	1	30	3	38	7

### Native American ancestry

To ensure that our method does not systematically underestimate Native American admixture due to the relatively small number of Native American reference individuals, we applied our method to 30 purely Native American individuals of Zapotecan ancestry from central Mexico collected as part of the Mexican Genome Diversity project (IMMEGEN; Silva-Zolezzi et al., 2009). Based on only 1957 overlapping markers all 30 individuals were classified as 100% Native American by our procedure using our proposed reference panel. Without denoising the native American admixture proportion of the 30 Zapotecan individuals had a mean of 0.989 and standard deviation of 0.016.

Another test of the accuracy of admixture estimates obtained via our proposed procedure involved 10 trios of Mexican origin (MEX) from the HapMap 3 dataset (Altshuler et al., 2010). Following basic laws of inheritance, a child should, on average, exhibit admixture proportions that are roughly equal to the average of his parents’ admixture proportions for the same continental populations. The deviations of the estimated admixture proportions obtained for the child from the averaged estimates of admixture proportions obtained for his or her parents reflect error associated with the estimates. We quantified these deviations and found that the average deviation (across the 10 trios used in this analysis) was 0.035, 0.009, and 0.02, 0.017 for European, African, Native American, and East Asian admixture respectively. (The average degree of admixture in the 20 parents was estimated to be 0.46, 0.05, and 0.46, 0.023 respectively).

## Discussion

Unsupervised model-based clustering methods implemented in STRUCTURE, ADMIXTURE or FRAPPE, as well as Principal Components Analysis (PCA)-based techniques can identify population structure in dense whole genome genotype data on a sample of individuals. However, these techniques pose a number of challenges; for example, model-based approaches often require *a priori* knowledge of the number of population clusters or subgroups in the data. Although techniques exist for estimation of the optimal number of clusters from the data, they are often based on heuristics, and may yield ambiguous results in certain cases. On the other hand, PCA as a data reduction technique requires an additional step of determining the optimal number of components to retain (Solovieff et al., 2010) even before clustering is undertaken, and may present a difficulty for more subtle ancestry determinations, e.g., within a continent (Johnson et al., 2011).

These issues can be overcome to some degree using various strategies and the results, e.g., clustering and PCA-based methods often agree (Patterson et al., 2006; Lawson et al., 2012). However, it can be difficult or impossible to determine the biogeographic ancestry of the individuals in each cluster without ”supervising” or including a comprehensive set of reference individuals with known ancestry in the analyses. Even when the ancestry of some individuals is known, clusters obtained via unsupervised clustering algorithms do not necessarily reflect grouping of individuals into actual known populations, but may instead capture relatedness or other artifacts contained within the dataset. We encountered this problem when attempting to cluster European, East Asian, African, and Native American individuals into four groups using ADMIXTURE during the construction of the reference panel. The resulting grouping was not consistent with the individuals’ documented ancestry. It is often of interest to determine, however, an individual’s ancestry in terms of the degree of contribution of a given set of populations. For this reason, we devised an approach that allows one to generate results that can be easily interpreted in the context of existing world populations. Additionally, these results can be compared across analyses involving various datasets, which is crucial to successfully replicating findings and performing meaningful meta-analyses. The procedure described here addresses global ancestry estimation, although it could easily be modified to determine the ancestry of a given chromosomal region by restricting the analysis to only those markers that reside within the region (Winkler et al., 2010). The total number of markers used in the reference panel, however, will limit the smallest size of the region for which ancestry estimation is feasible since a sufficient number of markers is needed to perform the estimation.

We assembled a reference panel by obtaining large publicly available genotype datasets and then, in a stepwise fashion, culled out a subset of individuals from this panel using combination of unsupervised and supervised clustering approaches that might be optimal for an ancestry determination associated with six continental populations. We note that the order of inclusion of the populations during the formation of the reference panel may impact the size of the reference populations included in the final panel, and this may, in turn, affect the estimated admixture proportions. We first incorporated populations that are genetically sufficiently differentiated with the goal of including data for as many available reference individuals as possible. Some processing and cleaning was, however, required, which led to the exclusion of data available for some individuals. We did this in such as way as to preserve the validity of the chosen individuals by meeting the following three conditions: (1) individuals in the reference panel do not exhibit ancestry from more than one continental population (e.g., individuals representing the European population do not have any East Asian ancestry); (2) the reference panel contains large enough sample of individuals from each continental population to minimize sampling bias; and, (3) individuals from samples representing each continental population are reasonably diverse to ensure that the population is well represented in terms of its population substructure (e.g., European population in a reference panel should be represented by individuals with Northern, Southern, Eastern, and Western European ancestry, Novembre et al., 2008). Of course, our efforts to fully satisfy these conditions were constrained by the availability of publicly available data. We therefore carried out a number of analyses to show that these conditions are sufficiently fulfilled.

We ensured that the first condition was satisfied by employing a stepwise clustering approach during the construction of the reference panel and imposing a minimum 90% membership rule for all added individuals (see Materials and Methods). This led to the exclusion of a number of samples. We further validated the condition via a resampling analysis. The clear separation of the individuals representing the various continental populations was also clearly apparent in the multidimensional scaling plot (see Figure 1). We tested the second and third condition in a specific set of circumstances by subjecting three sets of admixed individuals and one pure population of Native Americans to ancestry analyses using the reference panel. This last analysis showed that the ancestry of individuals of truly Native American ancestry (not represented in the reference panel) is correctly determined. This finding is important in the context of the last and perhaps most compelling validation study, in which we compared the admixture proportions of Mexican children to their expected admixture proportions based on their parents’ ancestry. If our proposed methodology is correct, then the deviation between expected and observed admixture proportions should be minimal and this was indeed the case. This result is encouraging as one of the source populations (Native Americans) is represented by only 47 individuals in the reference panel – the smallest group of individuals except for the Oceanic individuals. The fact that the deviation was quite small despite such a small reference sample lends credence to the accuracy of admixture estimates obtained at least for Hispanic, African American individuals, and perhaps individuals with Middle Eastern descent.

However, our results also show that admixture estimates computed using the outlined procedure contain some noise. This noise could result from several phenomena; for example, a small amount of noise can be explained by the non-deterministic nature of the algorithm implemented in ADMIXTURE. When we ran ADMIXTURE with two different seeds to determine admixture proportions in the 811 admixed individuals and compared the two sets of results, we observed an average error across all admixture proportions of only 0.0004. The highest error among the 811 times 6 (4866) estimates was 0.09. In this case, an individual was assigned a Central Asian admixture proportion of 0.09 and European admixture proportion of 0.91 in the first run using one seed, but was estimated to be 100% European in the second run using a different seed. Only 57 out of 4866 (1%) estimates of admixture proportions differed by more than 0.01. Consequently, when highly accurate admixture proportions are required, one may consider rerunning the analysis with a different seed and averaging the resulting admixture proportions. More sophisticated ensemble methods that can be used to aggregate results from several runs of ADMIXTURE using different seeds are described in, e.g., Breiman (1996).

Most noise in the admixture estimates is likely due to the sampling bias associated with our proposed reference panel. This limitation is given by the availability of genotype data in the public domain. Our study suggests that more data especially for Native American and Oceanic individuals should be added to the public domain. These two source populations moreover often contributed to the ancestry of many recently admixed individuals living in the United States. However, the presence of noise is especially evident for individuals from Central Asia. We hypothesize that this is due to the relatively higher genetic similarity between reference individuals from Europe and Central Asia in addition to their relatively high diversity possibly due to isolation by distance (Handley et al., 2007; see, e.g., Table 2). It is an interesting question for further research whether additional sampling of Central Asian individuals and their inclusion in the reference panel could reduce this noise.

In many applications, it is valuable to determine whether or not a given continental population contributed even a small degree of admixture to the overall genetic ancestry of an individual. This may for example be useful in efforts to determine local genetic ancestry at various genomic loci of an individual, for which all source populations that contributed to the individual’s ancestry must be known in advance (Chakraborty and Weiss, 1988; Stephens et al., 1994; McKeigue et al., 2000; Winkler et al., 2010). The proposed denoising procedure described in the Methods is intended to address this question, as well as reduce the noise in the estimated admixture proportions.

One obvious limitation of any approach that utilizes a panel of reference individuals with known ancestry is the fact that the design of the reference panel *a priori* defines the resolution or granularity of the ancestry assessment. For example, the reference panel proposed in this work is intended to be used to describe a target individual’s ancestry in terms of admixture proportions from six major continental populations. A different reference panel would need to be applied if one were interested in, e.g., admixture proportions for various European populations. This may be relevant as Price et al. (2008) showed that even European Americans are affected by population stratification bias.

## Conflict of Interest Statement

The authors declare that the research was conducted in the absence of any commercial or financial relationships that could be construed as a potential conflict of interest.
